# Adaptive density peak clustering based on Delaunay graph

**DOI:** 10.1371/journal.pone.0325161

**Published:** 2025-06-05

**Authors:** Wei Xingqiong, Li Kang

**Affiliations:** 1 School of Artificial Intelligence, Guangxi Minzu University, Nanning, China; 2 School of Physics and Electronic Information, Guangxi Minzu University, Nanning, China; UCAM: Universidad Catolica San Antonio de Murcia, CHINA

## Abstract

Clustering is a fundamental tool in data mining, widely used in various fields such as image segmentation, data science, pattern recognition, and bioinformatics. Density Peak Clustering (DPC) is a density-based method that identifies clusters by calculating the local density of data points and selecting cluster centers based on these densities. However, DPC has several limitations. First, it requires a cutoff distance to calculate local density, and this parameter varies across datasets, which requires manual tuning and affects the algorithm’s performance. Second, the number of cluster centers must be manually specified, as the algorithm cannot automatically determine the optimal number of clusters, making the algorithm dependent on human intervention. To address these issues, we propose an adaptive Density Peak Clustering (DPC) method, which automatically adjusts parameters like cutoff distance and the number of clusters, based on the Delaunay graph. This approach uses the Delaunay graph to calculate the connectivity between data points and prunes the points based on these connections, automatically determining the number of cluster centers. Additionally, by optimizing clustering indices, the algorithm automatically adjusts its parameters, enabling clustering without any manual input. Experimental results on both synthetic and real-world datasets demonstrate that the proposed algorithm outperforms similar methods in terms of both efficiency and clustering accuracy.

## 1. Introduction

Clustering is a fundamental unsupervised learning method in machine learning, widely applied in fields such as image recognition [1], information security [2], medical systems [3], bioinformatics [4], and pattern recognition [5]. It involves grouping data points into distinct clusters based on their features and similarity. Data points that are highly similar are placed in the same cluster, while those that are less similar are assigned to different clusters. However, defining the similarity between data points remains one of the key challenges in clustering [6].

Clustering algorithms are generally categorized into two types: hierarchical clustering and partition-based clustering [7]. In hierarchical clustering, the distance between data points determines cluster assignments, with clusters being formed either by progressively merging similar data points (agglomerative) or by dividing a single cluster into smaller ones (divisive). However, due to high computational complexity, hierarchical clustering is not suitable for large-scale, high-dimensional datasets, and it is also sensitive to noise and outliers, limiting its robustness [8].

In partition-based clustering, data is divided into groups without hierarchical structures. The algorithm selects initial cluster centers and then iteratively assigns data points to these clusters. These methods are often preferred in engineering applications due to their ability to efficiently represent and compress large datasets [9].

Density-based clustering algorithms, a subset of partition-based clustering, can detect clusters of arbitrary shapes. One such algorithm is Density-Based Spatial Clustering of Applications with Noise (DBSCAN) [10]. DBSCAN relies on two parameters: Minpts and Eps. Eps defines the neighborhood radius around a data point, and Minpts specifies the minimum number of neighbors required within that radius for a point to be considered a core point. Points with fewer neighbors are classified as noise. However, DBSCAN’s performance is sensitive to adjustments in Minpts and Eps, particularly with datasets of uneven density. Another prominent density-based algorithm is MeanShift [11], which, like DBSCAN, requires parameter tuning to achieve optimal performance. MeanShift also faces high computational complexity and may not yield satisfactory results on certain datasets.

Density Peak Clustering (DPC) is a novel density-based algorithm [12], based on two assumptions: first, high-density points are more likely to be cluster centers, and second, the distances between cluster centers are generally larger than those between other points. DPC uses local density and relative distances to create a decision graph, identifying cluster centers and assigning points to the nearest high-density clusters. However, DPC has some limitations, including its reliance on a manually defined cutoff distance for calculating local density, and it cannot automatically determine the number of clusters, requiring manual input.

To address these issues, various DPC variants have been proposed. Du et al. combined K-nearest Neighbors (KNN) with DPC to reduce computational complexity for high-dimensional datasets [13]. Liu et al. proposed a DPC variant based on Shared Nearest-Neighbor (SNN), using common neighbor relationships to calculate local density and mitigate the influence of the cutoff distance [14]. Yu et al. introduced a weighted local density sequence and a second-order assignment strategy to enhance DPC’s efficiency and accuracy [15]. Zhang et al. combined DPC with a density decay graph, allocating decay phenomena to each cluster and merging clusters through connection points to determine the final number of clusters [16]. Recent research also highlights the importance of local dominance in uncovering clusters within networks, further supporting the need for algorithms that do not rely on prior knowledge of parameters [17]. Additionally, studies on social perspectives of perceived distances have provided new insights into community structures, further emphasizing the importance of algorithms capable of detecting deeper structures within data [18].

Given the diversity and complexity of datasets, many clustering algorithms rely on prior information to uncover underlying structures. Thus, it is crucial for clustering algorithms to identify these structures without relying on manually set parameters. Unlike previous DPC variants, which inherently require the manual determination of a cutoff parameter, this paper proposes a DPC approach based on Delaunay triangulation. By constructing a Delaunay triangulation, we obtain correlation information between data points. Using the Validity Index for Arbitrary-Shaped Clusters based on Kernel Density Estimation (VIASCKDE) [19] as an internal index, we optimize connectivity among data points until the relevant conditions are met, allowing the algorithm to perform clustering without any dependence on manual input. Experimental results show that the proposed algorithm outperforms similar algorithms on both synthetic and real-world datasets.

This paper’s contributions are as follows:

(1) This paper proposes an innovative clustering method based on Delaunay triangulation to build adjacency relationships between data points, avoiding the manual setting of preset parameters such as cutoff distance. The clustering process is completely driven by the intrinsic structure of the data, eliminating the dependence on external parameters, making the algorithm more adaptive and flexible, and adaptable to complex data sets. Compared with traditional clustering algorithms that rely on manual settings (such as DBSCAN and DPC), this method automates the clustering process by dynamically determining the cluster center and number, solving the problem of manual parameter adjustment for multiple data sets.
(2) This paper proposes VIASCKDE as a new internal evaluation tool to optimize clustering quality and does not rely on metadata or label information of the data set. This method can effectively evaluate the clustering results without external labels by evaluating the intrinsic structure of the clusters, thus avoiding the dependence of traditional methods on real labels. Especially in practical applications with no or incomplete labels, VIASCKDE provides a more fair and unbiased clustering evaluation, effectively improving the universality of the algorithm.
(3) The algorithm is particularly good at processing data sets with irregular shapes and large density variations, and can automatically identify and adapt to complex data structures during the clustering process. Unlike traditional mean- or distance-based clustering methods (such as K-means), DPC-DG can identify clusters of arbitrary shapes and adapt to different density distributions, and is particularly suitable for highly complex data sets. By automatically adapting to the intrinsic structure of the data, DPC-DG provides more precise and robust clustering results, greatly improving the accuracy and applicability in practical applications.

The paper is structured as follows: Section 1 provides an introduction, Section 2 reviews related work, Section 3 details the proposed methodology, Section 4 describes the experiments and their results, and Section 5 concludes the paper, offering suggestions for future research directions.

## 2. Preliminaries

To address the issue of clustering algorithms relying on prior information, we propose a new method called Adaptive Density Peak Clustering based on Delaunay Graph (DPC-DG). The DPC-DG method consists of the following steps:

(1) Constructing a data correlation graph using Delaunay triangulation.
(2) Calculating the local density and average distance for each data point, followed by pruning the edges between points to effectively partition the clusters.
(3) Merging clusters through the VIASCKDE internal index.

### 2.1. DPC algorithm

DPC is an influential density-based clustering algorithm that postulates that the cluster centers of each cluster possess relatively high local densities and are situated far from other data points with high local densities. The DPC algorithm comprises several key steps: firstly, it calculates the local density for each data point. Secondly, it computes the distances between data points and constructs a decision graph based on these distances and local densities to identify the cluster centers. Finally, after determining the cluster centers, it assigns the remaining data points to their nearest cluster centers. The calculation of local density is represented by the following formula:


$$\rho_{i}=\sum_{j\neq i}\chi \left(d_{ij}-d_{c}\right)$$ (1)

$$\chi (x)=\left\{ \begin{array}{c} 1,\;x<0\\ 0,\;x\geq 0 \end{array}\right.\;$$ (2)

Here, $d_{ij}$ represents the Euclidean distance between data point $x_{i}$ and data point $x_{j}$, while $d_{c}$ denotes the cutoff distance. The recommended value for $d_{c}$ is typically 1% to 2% of the sum of all pairwise distances between data points. For small sample datasets, a Gaussian kernel function can also be employed to calculate local density, which is represented by the following formula:


$$\rho_{i}=\sum_{j\neq i}e^{-{\left(\frac{d_{ij}}{d_{c}}\right)}^{2}}$$ (3)

After calculating the local density of each data point, the distances between data points need to be computed. This is represented by the following formula:


$$\begin{array}{c} \delta_{i}=\left\{ \begin{array}{c} \min_{j}{(d}_{ij}),\;if\;\exists \;j.s.t\;\rho_{j}>\rho_{i\;}\\ \max_{j}{(d}_{ij}),\;otherwise\; \end{array}\right.\; \end{array}$$ (4)

Based on the above concepts, if the local density value of data point $x_{i}$ is the maximum, then the corresponding distance $\delta_{i}$ is the farthest distance from $x_{i}$ to any other data point. Otherwise, $\delta_{i}$ for data point $x_{i}$ is the distance to its nearest higher-density data point. DPC generates a decision graph using both local density and distance, with the cluster centers identified based on the highest local densities and distances. Data points with low local densities and relatively large distances are considered noise, and the remaining data points are assigned to the nearest cluster center.

### 2.2. VIASCKDE index

Clustering indices can be classified into internal and external indices. External indices require the true labels of the dataset for comparison, such as Purity [18], Rand Index [20], Adjusted Rand Index [21], and Normalized Mutual Information [22]. These indices rely on the availability of true labels to assess clustering performance.

In contrast, internal indices do not require true labels. Instead, they evaluate clustering based on the separation and compactness of data points. Examples include the Silhouette Index (SI) [23], Dunn Index (DI) [24], Davies-Bouldin Index (DB) [25], and Calinski-Harabasz Index (CH) [26]. These indices focus on the geometry of the clusters themselves and can be used even when true labels are not available.

VIASCKDE is a novel internal index introduced in this paper. Unlike traditional indices, VIASCKDE remains unaffected by the shape of the clusters, making it suitable for detecting clusters of arbitrary shapes found in complex datasets. Kernel Density Estimation (KDE) is employed within VIASCKDE to assess how well data points are distributed and whether they are correctly positioned within their clusters. By assigning higher weight to points in dense regions, VIASCKDE minimizes the impact of cluster shape and focuses on assessing the compactness and separation of the clusters. This ability to weight points based on density allows for more accurate and unbiased evaluation of the clustering performance. To perform clustering optimization iteratively, VIASCKDE utilizes an average distance check that focuses on both compactness and separation during the iterative clustering process. This allows the algorithm to dynamically adjust cluster relationships based on their local densities, improving the overall clustering quality in each iteration. The first step is to construct the Delaunay triangulation for the dataset, connecting data points into a graph based on their proximity. The edges of this triangulation represent the relationships between data points and form the base for calculating local density. This graph provides essential information for adjusting cluster boundaries. KDE is applied to estimate the density around each data point. The density function assigns a higher weight to points in denser regions, ensuring that the clustering process focuses on areas with higher data concentrations. By considering local density, VIASCKDE helps to adapt the clustering process to regions with varying data distributions. Once the local density is determined, the next step is to compute the compactness and separation for each data point. Compactness refers to how close points within the same cluster are to each other, while separation measures how distinct a cluster is from others. VIASCKDE calculates the Compactness and Separation Value (COSED), which is given by the following formula:


$$COSED(x)=W_{KDE}\frac{b(x)-a(x)}{\max\left\{a(x),b(x)\right\}}$$ (5)

$$a(x)=\min_{x\in C_{i},y\in C_{i}}\left\{|x\;-\;y{|}^{2}\right\}$$ (6)

$$b(x)=\min_{x\in C_{i},y\in C_{j},x\neq y}\left\{|x\;-\;y{|}^{2}\right\}$$ (7)

Here, $W_{KDE}$ represents a non-parametric probability density function, while a(x) denotes the compactness of a data point, which is the minimum distance from the data point to its assigned cluster center. On the other hand, b(x) represents the separation of a data point, which is the minimum distance from the data point to the nearest cluster center belonging to a different cluster. $\;C_{i}$ and $C_{j}$ indicate the cluster assignments for data points $x_{i}$ and $x_{j}$ respectively.

After the initial COSED calculations, the algorithm iterates to adjust the relationships between data points based on their compactness and separation. In each iteration:Points with low compactness are moved towards their cluster center. Points with high separation are assigned to clusters that are farther apart, improving the cluster boundaries. This iterative process refines the clusters until the best compactness and separation values are achieved, ensuring that clusters with complex shapes and varying densities are accurately detected. Once the iterative adjustments are completed, the Compactness and Separation Value of a Cluster (COSEC) is calculated for each cluster:


$$COSEC\left(C_{i}\right)=\frac{1}{n}\sum_{i=1}^{n}COSED\left(x_{i}\right)$$ (8)

where COSEC represents the mean sum of COSED values within the current cluster, and n is the total number of data points in cluster $C_{i}$.

Finally, the overall clustering quality is measured by VIASCKDE, which aggregates the *COSEC* values across all clusters, weighted by the number of points in each cluster:


$$VIASCKDE=\frac{\sum_{j=1}^{k}n_{j}COSEC\left(C_{i}\right)}{\sum_{j=1}^{k}n_{j}}$$ (9)

where *k* is the number of clusters, $n_{j}$ is the number of data points in cluster $C_{j}$, and the value of COSEC for each cluster is calculated using the formula mentioned in (8) or a similar formula that reflects the compactness and separation within that cluster.

In summary, the VIASCKDE index iteratively performs an average distance check to assess compactness and separation, optimizing the clustering process. By adjusting the cluster relationships based on local density and distance checks, VIASCKDE ensures that clusters are both tightly grouped and well-separated. This iterative refinement makes DPC-DG an adaptive and efficient clustering method, able to handle arbitrary-shaped clusters and datasets with varying densities, all without requiring manual parameter tuning.

## 3. Adaptive density peak clustering based on Delaunay graph (DPC-DG)

The proposed DPC-DG method introduces significant improvements over traditional clustering algorithms by offering automatic parameter selection and enhanced adaptability. Unlike previous methods that require manual tuning of parameters like cutoff distances and cluster numbers, DPC-DG dynamically adjusts these parameters based on the data’s intrinsic structure. This makes the algorithm more flexible and better suited for datasets with varying densities and complex structures.

### 3.1. Constructing the delaunay triangulation

Delaunay triangulation is a widely used method in computational geometry for partitioning multidimensional datasets into smaller units such as triangles (in 2D) or tetrahedra (in 3D). The goal of Delaunay triangulation is to create a set of triangles that maximize the minimum interior angle, avoiding the formation of narrow triangles. This method partitions the space by connecting data points in such a way that no data point lies inside the circumcircle of any triangle in the partition.

In DPC-DG, we first map the dataset into a neighborhood graph where the edges between data points represent their connectivity. Delaunay triangulation is employed to construct similarity matrices within this neighborhood graph. A Delaunay graph consists of non-overlapping, connected triangles, where the circumcircle of each triangle does not contain any other points inside it. This graph effectively captures the connectivity between data points, ensuring that the connections respect the inherent geometric structure of the data, which is crucial for clustering datasets with varying density distributions.

**Fig 1:**
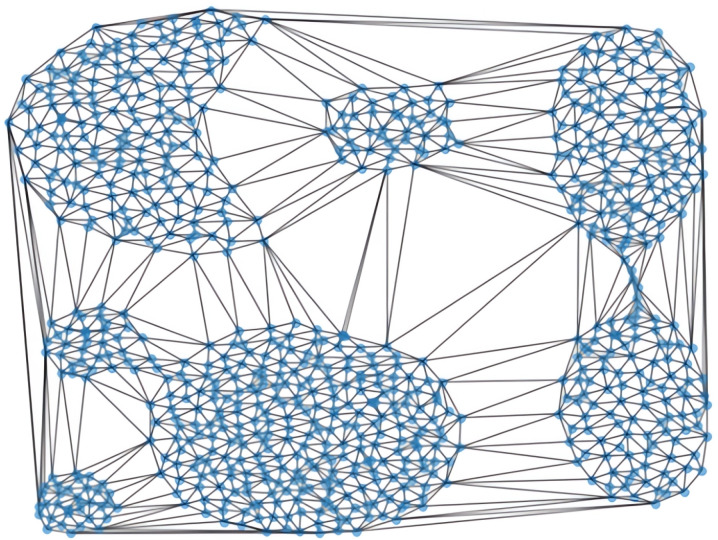
The Delaunay triangulations of aggregation dataset.

By using Delaunay triangulation, the connectivity between data points is encoded in a graph where each edge represents a local relationship. The Delaunay graph thus helps in revealing the global and local distribution of data points, as shown in Fig 1.

Key Steps in Constructing Delaunay Triangulation:

(1) Each point in the dataset is treated as a node in the graph.
(2) Triangles are formed by connecting points such that no other points fall within the circumcircle of each triangle.
(3) The resulting graph shows the local connectivity of data points, which is used in subsequent steps for partitioning and clustering.

**Fig 2:**
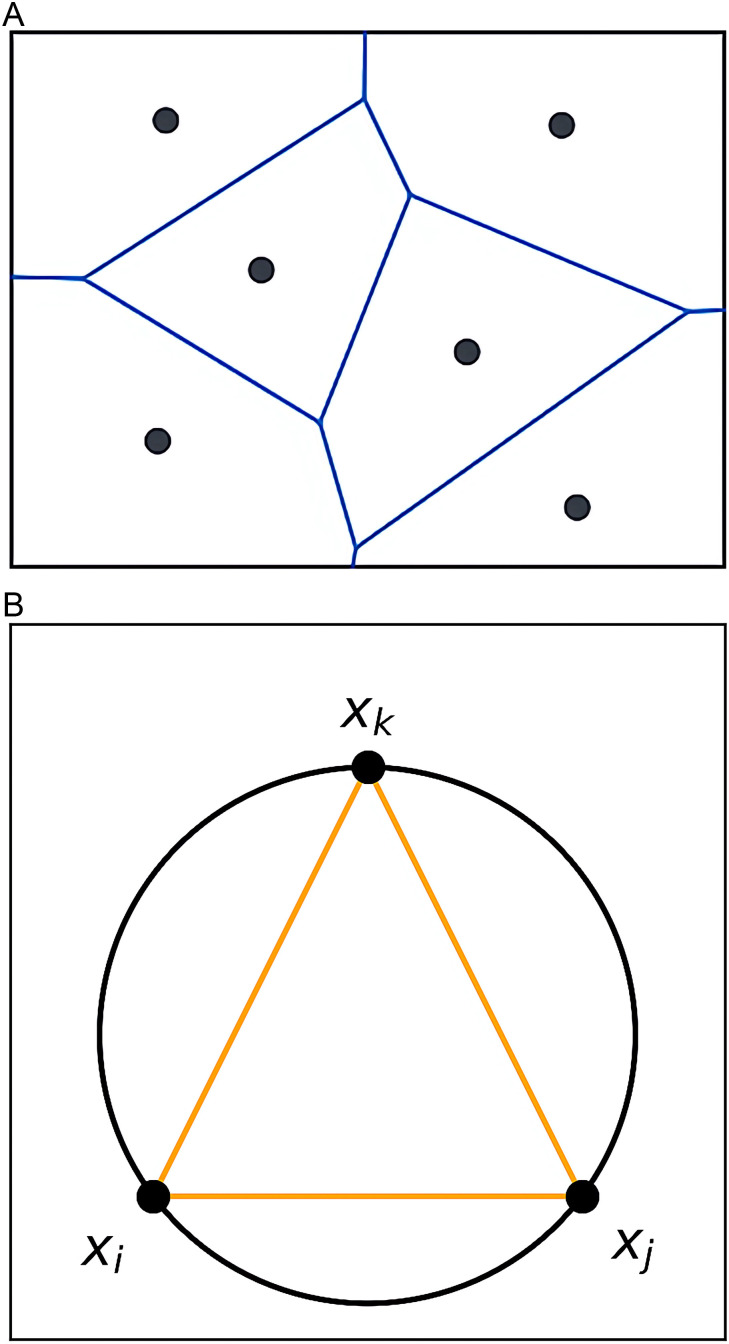
Example of Delaunay triangulation. **(a)** Partitioning the dataset into multiple unit grids. **(b)** Interconnecting data points within the unit grids.

The Delaunay triangulation assumes that there are N finite data points $X=\left\{x_{1},x_{2},x_{3},\cdots ,x_{n}\right\}$ in the dataset, as shown in Fig 2(a), where the dataset X is partitioned into N unit grid forms. For each data point in the dataset X, if there exist three data points $x_{I},x_{j},x_{k}\in X$ that can form a circumcircle without any other data points inside it, these three data points are interconnected to form a triangle, as illustrated in Fig 2(b).

### 3.2. Partitioning clusters

The goal of partitioning clusters is to determine the boundaries between clusters based on the connectivity between data points. Using the Delaunay graph, we compute the average distance between each point and its neighboring points to build a clear understanding of the structure of the dataset.

To calculate the average distance between a data point $x_{i}$ and its neighbors, we use the following formula:


$$meandist\left(x_{i}\right)=\frac{1}{n_{adj}}\sum_{j\in neighbors(x_{i})}{\left|x_{i}-x_{j}\right|}^{2}$$ (10)

**Fig 3:**
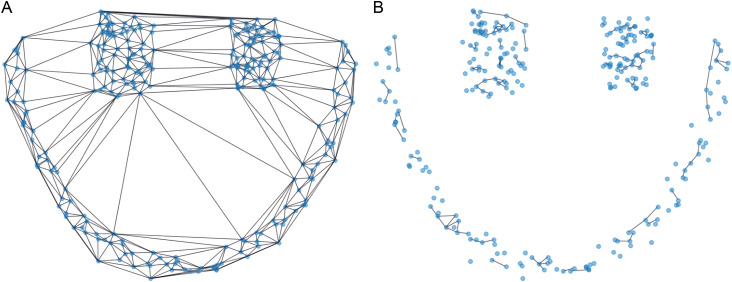
The compared Delaunay triangulations of cutting edges. **(a)** Edges of data points before pruning. **(b)** Edges of data points after pruning.

Where $n_{adj}\;$ represents the total number of neighbors connected to the data point $x_{i}$, and$\;\left|x_{i}-x_{j}\right|$ is the Euclidean distance between data points $x_{i}$ and $x_{j}$. Once the average distance is calculated, edges between points are evaluated. If the distance between a point $x_{i}$ and any of its neighbors $x_{j}$ is greater than half of the average distance, the edge between them is pruned. This step removes less significant connections, ensuring that only the most meaningful relationships (those representing true clusters) are retained. This pruning process ensures that the graph is divided into distinct clusters, as shown in Fig 3. The pruning step allows the algorithm to partition the dataset into isolated data points and meaningful clusters.

### 3.3. Merging clusters

The local density of each data point is calculated using the following formula:


$$\rho_{i}=\sum_{j\neq i}e^{-{\left(\frac{d\left(x_{i},x_{j}\right)}{meandist\left(x_{i}\right)}\right)}^{2}}$$ (11)

where $d\left(x_{i},x_{j}\right)$ is the Euclidean distance between data points $x_{i}$ and $x_{j}$, and *meandist(*$x_{i}$*)* is the average distance of $x_{i}$ ‘s neighbors. Data points with zero edges are identified as isolated points, whereas data points with non-zero edges are considered as belonging to clusters. The isolated data points are then assigned to the nearest high-density data point, leading to the formation of distinct clusters.

**Fig 4:**
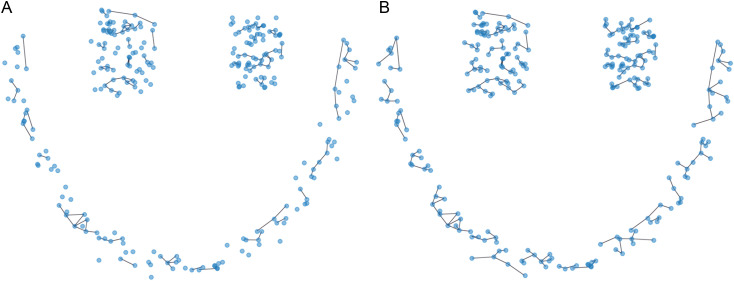
Comparison of Delaunay triangulations after assigning data points. **(a)** Unassigned data points. **(b)** After assigning data points.

Data points with zero edges are identified as isolated data points, whereas data points with non-zero edges are considered as belonging to clusters. The isolated data points are then assigned to the nearest high-density data point, leading to the formation of distinct clusters. This process is illustrated in Fig 4 below.

The categorization of data points can be formally expressed as:


$$data\;point=\left\{ \begin{array}{c} isolated\;point,ifedge\left(x_{i}\right)=0\\ clusters,\;if\;edge\left(x_{i}\right)>0 \end{array}\right.\;$$ (12)

Here,$\;edge\left(x_{i}\right)$ represents the number of edges connecting data point $x_{i}$ to other data points.

After the completion of cluster partitioning, as shown in Fig 4(b), we can observe that many highly dense and interconnected data points are independently dispersed, which can be considered as separate clusters. Before merging the clusters, we first identify the core data points within each cluster as those having more than one edge (i.e., $edge\left(x_{i}\right)$ > 1). These core data points typically reside in the dense regions of the cluster. If a data point has only one edge, it is likely to be located on the edge of the cluster.

The categorization of cluster points can be formalized as follows:


$$cluster\;point=\left\{ \begin{array}{c} core\;point,\;ifedge\left(x_{i}\right)>1\\ border\;point,\;if\;edge\left(x_{i}\right)=1 \end{array}\right.\;$$ (13)

**Fig 5:**
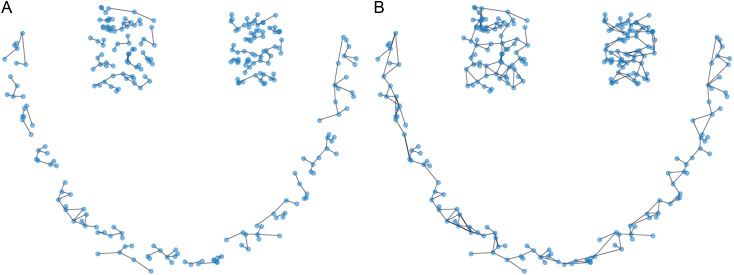
Comparison of Delaunay triangulations before and after merging clusters. **(a)** Unmerged Clusters. **(b)** After Merging Clusters.

Next, we identify the core data points of each cluster and calculate the distances between these core points of different clusters. We then determine if the distance between the core points of two clusters is less than the current average distance. If so, we connect these two data points. Using the VIASCKDE internal index, we iteratively perform this average distance check and proceed with merging clusters based on these conditions. This process is illustrated in Fig 5.

### 3.4. Time complexity

The DPC-DG algorithm consists of three main parts:

(1) **Constructing the Delaunay Triangulation Graph of Data Points:** The time complexity of this part is $O\left(n^{2}\right)$, where n represents the number of data points. This involves creating the Delaunay triangulation matrix and graph for each data point.
(2) **Clustering Partition**: This part computes the local density of each data point using Equation (11) and the average distance using Equation (10). Based on the average distance, it decides whether to remove edges between data points, thereby partitioning the data into distinct clusters. The time complexity of this part is also $O\left(n^{2}\right)$.
(3) **Assigning Isolated Data Points to Clusters**: In this part, isolated data points (those with no edges) are identified and assigned to the nearest cluster. The time complexity for this assignment is $O\left({n_{s}}^{2}\right)$, where $n_{s}$ is the number of isolated data points. Additionally, the merging of clusters through iterative application of the VIASCKDE internal index has a time complexity of $O\left(kn^{2}\right)$, where *k* is the number of iterations and *n* is the total number of data points in the dataset.

**Overall Time Complexity of DPC-DG:**

Combining these parts, the total time complexity of DPC-DG is $O\left(n^{2}\right)$+$\;O\left(n^{2}\right)$+$\;O\left({n_{s}}^{2}\right)$+$\;O\left(kn^{2}\right)$. Since $n_{s}\leq n$ and in practice, k is often a small constant relative to n, the dominantterm is $O\left(kn^{2}\right)$.

Pseudo-code for DPC-DG:

Algorithm 1: Pseudo-code for DPC-DG

**Input:** Dataset D

**Output:** Cluster labels

1. Compute the Delaunay triangulation matrix for each data point in D

2. Construct the Delaunay triangulation graph based on the matrix

3. Use Equations (10) and (11) to compute average distance and local density for each data point

4. Based on whether the distance between data points is above their respective average distance, decide to remove edges

5. Identify isolated data points and clusters using Equation (12)

6. Assign isolated data points to the nearest cluster

7. Iteratively merge clusters using VIASCKDE until the stopping condition is met

8. Return the clustering results

## 4. Experimental results and analysis

In this paper, we evaluate the performance of DPC-DG using both synthetic datasets with varying shapes and dimensions, as well as real-world datasets from the UCI repository. Table 1 provides detailed information about these datasets. The clustering results of DPC-DG are compared with several widely used clustering algorithms, including DPC [12], K-means [27], HDBSCAN [28], Gaussian Mixture Model (GMM) [29], Fuzzy C-means (FCM) [30], Spectral Clustering (SC) [31], Affinity Propagation (AP) [32], DPCSA [15] and LW-DPC [33].To evaluate the clustering performance, we use five commonly employed clustering indices: Accuracy (ACC) [34]: Measures the proportion of correctly classified instances, a higher value indicates better clustering accuracy. Adjusted Rand Index (ARI) [21]: This metric adjusts for chance groupings and compares the agreement between predicted and true clusters, ranging from −1 (no agreement) to 1 (perfect agreement), higher values indicate better clustering results. Normalized Mutual Information (NMI) [22]: This measures the amount of information shared between the predicted and true labels, a higher value indicates that the algorithm’s clusters align more closely with the true structure. Adjusted Mutual Information (AMI) [35]: Similar to NMI, but adjusted for chance, higher values indicate better alignment with true clusters. and Fowlkes-Mallows Index (FMI) [36]: Measures the geometric mean of precision and recall, a higher value indicates better clustering performance. For each index, a value closer to 1 indicates better performance. Additionally, we apply the Wilcoxon signed-rank test, a non-parametric statistical test [37], to assess the significance of the differences between the clustering results of the algorithms. The FMI is used as the test statistic to compare DPC-DG with K-means, HDBSCAN, DPC, GMM, FCM, Meanshift, SC, and AP. All experiments are conducted in Python 3.6 on a machine running Windows 10, with an AMD Ryzen 5 2500U @ 2GHz processor and 8GB of RAM. The parameter settings for comparative algorithms are shown in Table 2.

**Table 1:** The details of experiment datasets.

Dataset	Instances	Dimensions	Cluster
***Synthetic Data***			
Aggregation	788	2	7
Compound	399	2	6
R15	600	2	15
Four Lines	511	2	4
Smile	266	2	3
Circle	299	2	3
***Real-world Data***			
German	1000	24	2
Liver	345	6	2
Thyroid	215	5	3
DIM1024	1024	1024	16
Landsat	2000	36	6
Pima	768	8	2
Spambase	4601	57	2
WPBC	198	33	2

**Table 2:** Parameter Settings for Comparative Experiments.

Algorithm	Parameter	Detailed description
DPC-GP	K = 2 ∼ 50	Number of neighbors
DPC	𝑑_𝑐_ = 2% ~ 4%	Cut off distance
K-means	*n*_c_ = 2 ~ 15	Number of clusters
	Iteration = 300	number of iterations
DBSCAN	Minpts = 2 ∼ 20	Sample threshold
	ε = 0.01 ∼ 0.5	Data point radius
GMM	Iteration = 200	number of iterations
HDBSCAN	Min_size = 2 ∼ 20	Cluster size
FCM	*μ* = 2	Fuzzy index
	Iteration = 300	number of iterations
Spectral Clustering	𝑛_𝑐_ = 3 ~ 15	Number of clusters
Affinity Propagation	Iteration = 300	number of iterations
DPCSA	𝑛_𝑐_ = 2 ~ 15	Number of clusters
LW-DPC	K = 2 ∼ 50	Number of neighbors

### 4.1. Experiments on synthetic data

**Fig 6:**
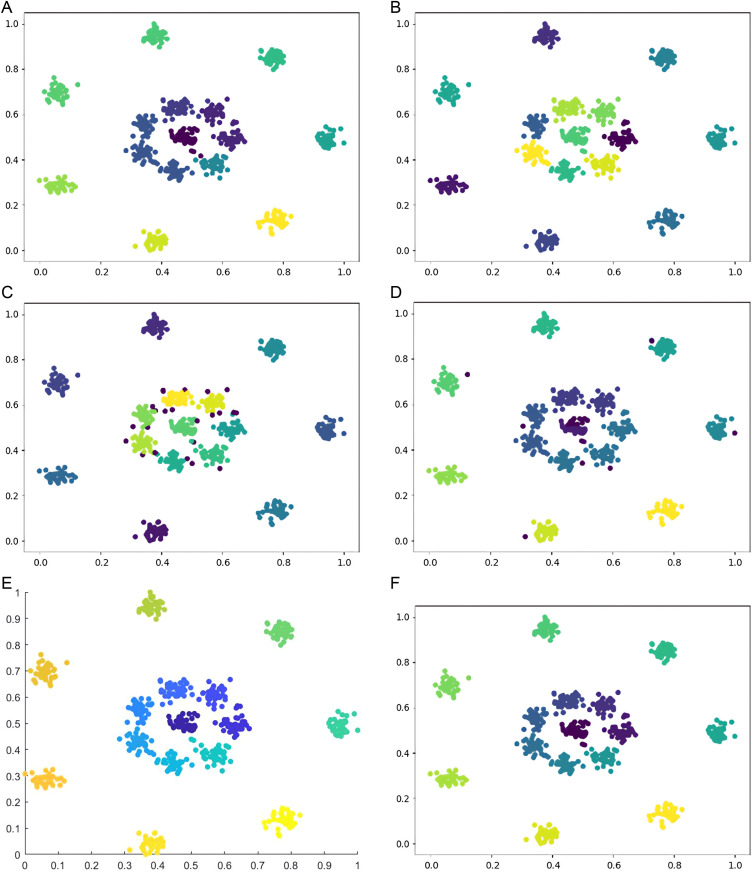
Visual Clustering Results of Algorithms on R15 Dataset. **(a)** DPC-DG, **(b)** K-means, **(c)** HDBSCAN, **(d)** DPC, **(e)** DPCSA, **(f)** AP.

**Fig 7:**
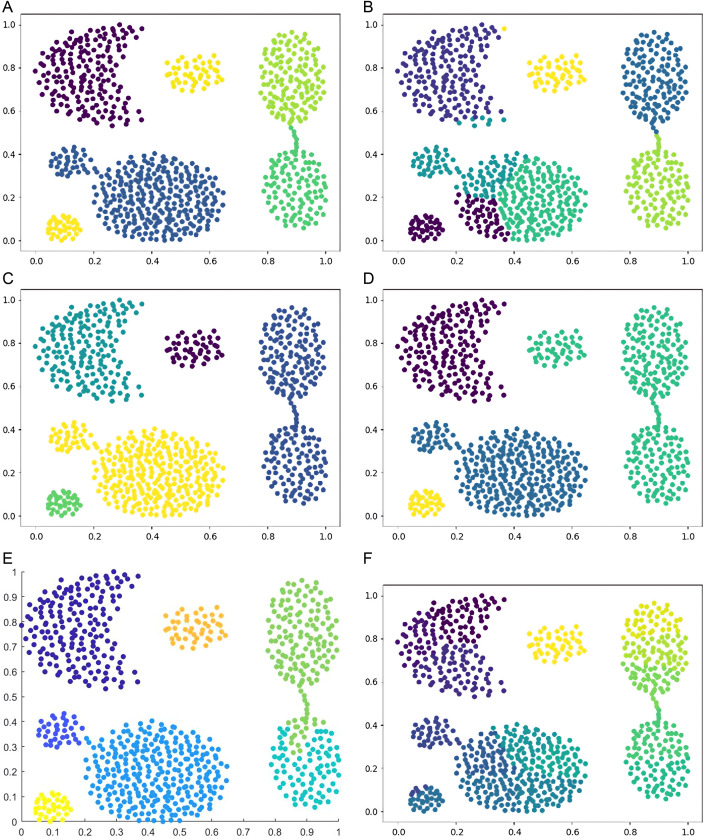
Visual Clustering Results of Algorithms on Aggregation Dataset. **(a)** DPC-DG, **(b)** K-means, **(c)** HDBSCAN, **(d)** DPC, **(e)** DPCSA, **(f)** AP.

**Fig 8:**
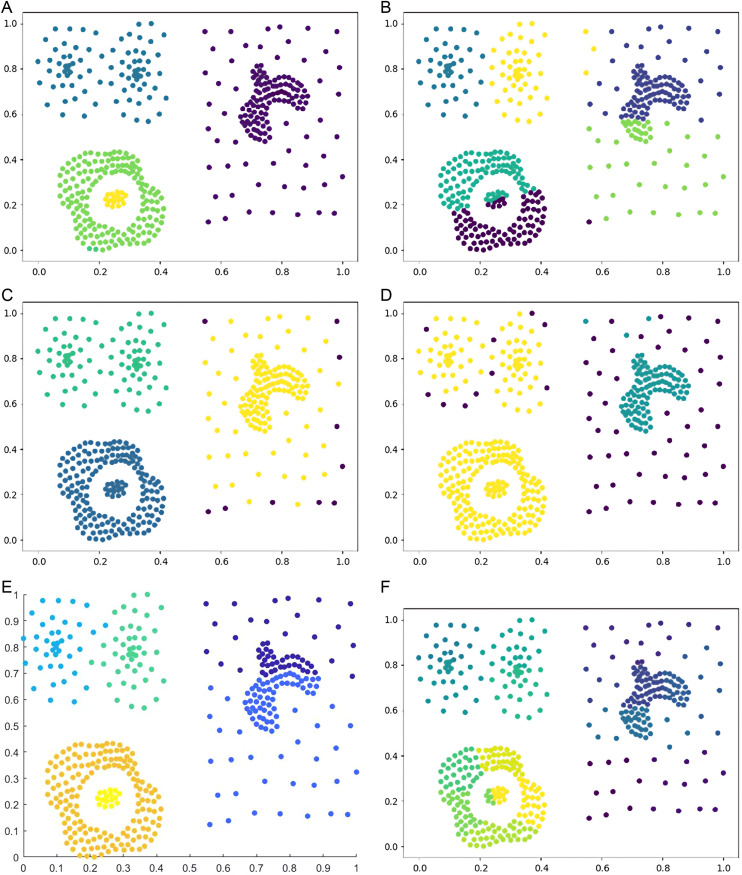
Visual Clustering Results of Algorithms on Compound Dataset. **(a)** DPC-DG, **(b)** K-means, **(c)** HDBSCAN, **(d)** DPC, **(e)** DPCSA, **(f)** AP.

**Fig 9:**
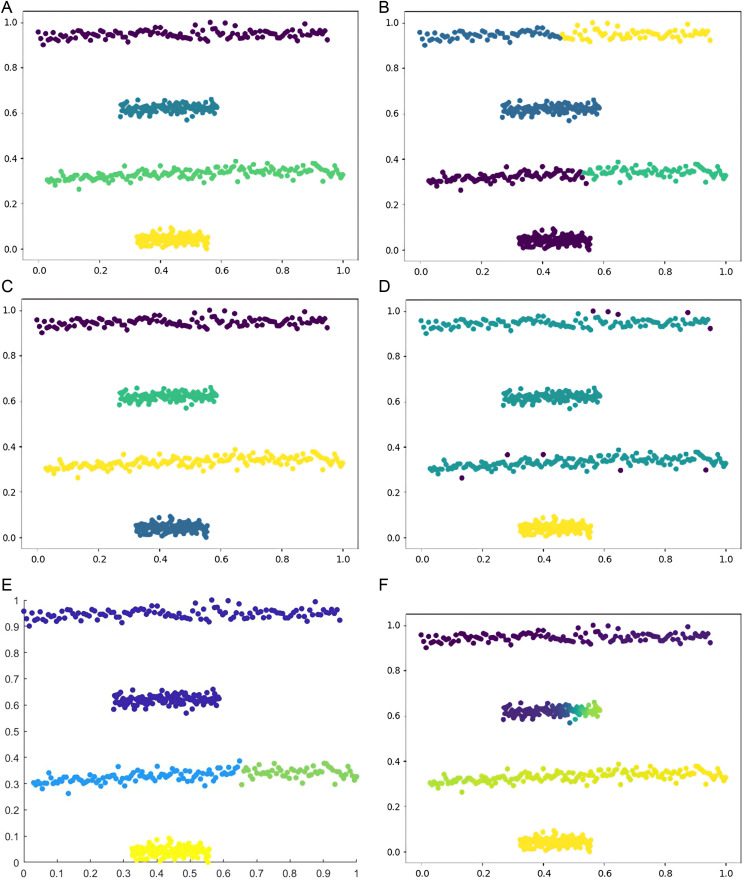
Visual Clustering Results of Algorithms on Four Lines Dataset. **(a)** DPC-DG, **(b)** K-means, **(c)** HDBSCAN, **(d)** DPC, **(e)** DPCSA, **(f)** AP.

**Fig 10:**
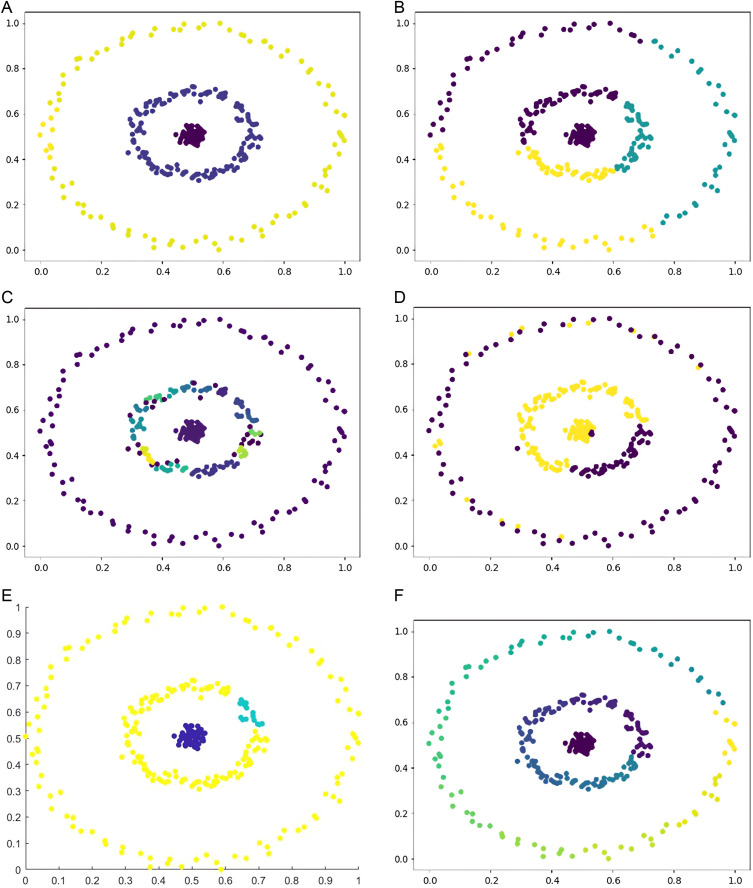
Visual Clustering Results of Algorithms on Circle Dataset. **(a)** DPC-DG, **(b)** K-means, **(c)** HDBSCAN, **(d)** DPC, **(e)** DPCSA, **(f) A.**

**Fig 11:**
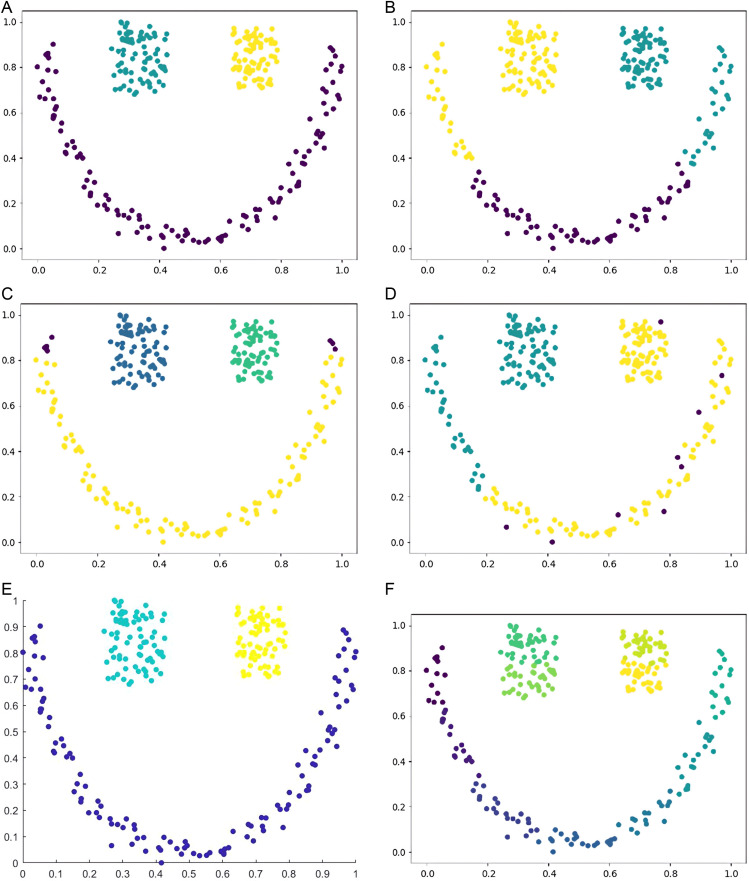
Visual Clustering Results of Algorithms on Smile Dataset. **(a)** DPC-DG, **(b)** K-means, **(c)** HDBSCAN, **(d)** DPC, **(e)** DPCSA, **(f)** FCM.

Six types of synthetic datasets are employed to evaluate the performance of DPC-DG. Figs 6–11 visually demonstrate the clustering results of DPC-DG in comparison with other algorithms, while Table 3 summarizes the experimental outcomes of DPC-DG and its counterparts on these six synthetic datasets.

**Table 3:** Clustering Results of Algorithms on Synthetic Datasets.

Algorithms	R15					Aggregation				
	ACC	ARI	NMI	AMI	FMI	ACC	ARI	NMI	AMI	FMI
DPC-DG	0.8	0.76	0.93	0.93	0.8	0.95	0.91	0.94	0.93	0.93
K-means	**1**	**0.99**	**0.99**	**0.99**	**0.99**	0.85	0.72	0.83	0.83	0.78
HDBSCAN	0.96	0.94	0.95	0.95	0.95	0.83	0.81	0.89	0.89	0.87
DPC	0.70	0.69	0.89	0.88	0.74	0.77	0.73	0.83	0.83	0.82
GMM	0.70	0.64	0.83	0.82	0.68	0.78	0.81	0.90	0.89	0.86
FCM	0.36	0.33	0.63	0.61	0.46	0.77	0.72	0.78	0.78	0.8
DPCSA	0.99	0.98	0.98	0.98	0.98	**0.98**	**0.96**	**0.96**	**0.96**	**0.98**
SC	0.67	0.61	0.84	0.83	0.67	0.75	0.58	0.77	0.77	0.67
AP	**1**	0.99	0.99	0.99	0.99	0.39	0.37	0.72	0.71	0.52
LW-DPC	**1**	0.95	0.92	0.89	0.94	0.92	0.88	0.94	0.92	0.85
**Algorithms**	**Compound**					**Four Lines**				
	**ACC**	**ARI**	**NMI**	**AMI**	**FMI**	**ACC**	**ARI**	**NMI**	**AMI**	**FMI**
DPC-DG	0.76	**0.79**	**0.85**	**0.84**	**0.85**	**1**	**1**	**1**	**1**	**1**
K-means	0.68	0.56	0.72	0.71	0.66	0.73	0.51	0.68	0.68	0.65
HDBSCAN	0.76	0.75	0.80	0.80	0.83	1	1	1	1	1
DPC	0.74	0.53	0.65	0.65	0.71	0.53	0.32	0.55	0.55	0.62
GMM	0.66	0.62	0.75	0.75	0.71	0.57	0.33	0.42	0.42	0.50
FCM	0.58	0.51	0.67	0.66	0.63	0.63	0.38	0.52	0.51	0.54
DPCSA	**0.80**	0.76	0.80	0.80	0.82	0.67	0.64	0.80	0.80	0.81
SC	0.49	0.27	0.48	0.47	0.43	0.75	0.47	0.67	0.67	0.63
AP	0.36	0.3	0.63	0.61	0.45	0.22	0.15	0.48	0.36	0.33
LW-DPC	0.76	0.73	0.81	0.76	0.81	0.98	0.96	0.95	0.89	0.92
**Algorithms**	**Circle**					**Smile**				
	**ACC**	**ARI**	**NMI**	**AMI**	**FMI**	**ACC**	**ARI**	**NMI**	**AMI**	**FMI**
DPC-DG	**0.97**	**0.96**	**0.95**	**0.95**	**0.97**	**1**	**1**	**1**	**1**	**1**
K-means	0.46	0.06	0.17	0.16	0.40	0.80	0.49	0.59	0.58	0.67
HDBSCAN	0.63	0.51	0.61	0.58	0.67	1	0.95	0.96	0.96	0.97
DPC	0.59	0.20	0.23	0.23	0.55	0.58	0.34	0.45	0.45	0.61
GMM	0.65	0.51	0.67	0.66	0.73	0.50	0.25	0.45	0.44	0.56
FCM	0.46	0.06	0.07	0.06	0.41	0.87	0.65	0.70	0.69	0.77
DPCSA	0.62	0.39	0.63	0.63	0.65	1	1	1	1	1
SC	0.45	0.05	0.16	0.16	0.4	0.79	0.47	0.58	0.58	0.66
AP	0.33	0.27	0.56	0.54	0.47	0.38	0.35	0.63	0.62	0.54
LW-DPC	0.87	0.85	0.83	0.92	0.93	0.88	0.95	0.97	0.86	0.95

From Table 3 and Figs 6-11, we observe that DPC-DG outperforms other algorithms on most datasets, especially in terms of ACC, ARI, and FMI. Specifically, on the R15 dataset, K-means obtains the highest ACC and ARI scores, indicating that K-means is effective when clusters are well separated and similar in shape. However, DPC-DG performs better in NMI and AMI, demonstrating its robustness in handling more complex cluster shapes. On the Aggregation dataset, detecting cluster centers in arbitrary-shaped clusters is crucial, and DPC-DG performs well by identifying cluster centers through local density peaks. This is a significant improvement over K-means and other partition-based algorithms, which have difficulty detecting such centers. The Silhouette score and ARI further confirm that DPC-DG provides more accurate and meaningful clustering in this case. On the Compound and Four Lines datasets, DPC-DG demonstrates its ability to handle different densities and complex cluster shapes, outperforming K-means and FCM, which perform poorly on datasets with different densities. On the Circle and Smile datasets, which involve diverse data distributions, DPC-DG achieves perfect clustering with ACC, ARI, NMI, and FMI scores all equal to 1. In contrast, algorithms such as K-means and SC fail to correctly identify clusters, resulting in severe misassignments. These results clearly demonstrate that DPC-DG can effectively identify clusters with arbitrary shapes and densities, outperforming other clustering algorithms in most cases. While DPC-DG performs well on low-dimensional synthetic datasets, its performance may degrade in high-dimensional datasets due to the “curse of dimensionality.” In high-dimensional spaces, Delaunay triangulation may result in less accurate adjacency relationships, leading to misclassification, especially when data points are sparse. The domino effect may also cause adjacent data points to be incorrectly assigned to different clusters. Despite these challenges, DPC-DG remains competitive in identifying patterns in high-dimensional data, especially when combined with powerful internal evaluation metrics such as the Adjusted Rand Index (ARI) and the Silhouette score, which help mitigate the negative impact of high dimensionality on clustering performance. In our experiments, we used a combination of external and internal evaluation metrics. For datasets with known labels, external metrics such as NMI and FMI were used to evaluate how well the algorithm matches the true clustering structure. Internal metrics such as ARI and Silhouette score were used to evaluate the clustering quality without relying on the true labels. These metrics consistently show that DPC-DG outperforms other clustering algorithms in terms of clustering quality, especially on synthetic datasets with complex shapes and varying densities. This confirms that DPC-DG produces clustering results that are not only superior in accuracy but also statistically different from other clustering methods.

### 4.2. Experiments on real-world data

Real-world data is used to test the performance of algorithms in high-dimensional spaces and real environments. We used six UCI real-world datasets to evaluate the performance of the algorithm. The clustering results of the DPC-DG algorithm and the comparison algorithm on the real-world data are shown in Table 4.

**Table 4:** Clustering results of real datasets.

Algorithms	German					Liver				
	ACC	ARI	NMI	AMI	FMI	ACC	ARI	NMI	AMI	FMI
DPC-DG	**0.68**	0.02	0.01	0.01	**0.73**	**0.55**	0.01	0.00	0.00	**0.68**
K-means	0.64	**0.05**	0.01	0.01	0.01	0.55	0.01	0.00	0.00	0.63
HDBSCAN	0.66	0.01	0.02	0.01	0.69	0.53	0.02	0.01	0.00	0.60
DPC	0.66	0.03	0.02	0.01	0.71	0.54	**0.04**	**0.04**	**0.04**	0.53
GMM	0.64	0.01	0.00	0.00	0.66	0.05	0.01	0.01	0.01	0.58
FCM	0.60	0.04	0.04	**0.04**	0.56	0.51	0.00	0.00	0.00	0.53
DPCSA	0.66	0.00	0.03	0.02	0.72	0.46	0.00	0.00	0.00	0.54
SC	0.60	0.00	0.00	0.00	0.66	0.50	0.00	0.01	0.01	0.68
AP	0.04	0.00	**0.04**	0.03	0.10	0.09	0.00	0.04	0.01	0.15
LW-DPC	0.66	0.00	0.01	0.00	0.69	0.45	0.02	0.00	0.01	0.59
**Algorithms**	**Landsat**					**Pima**				
	**ACC**	**ARI**	**NMI**	**AMI**	**FMI**	**ACC**	**ARI**	**NMI**	**AMI**	**FMI**
DPC-DG	**0.70**	**0.55**	**0.61**	0.59	**0.65**	**0.65**	0.01	0.00	0.00	**0.71**
K-means	0.68	0.52	0.61	**0.61**	0.61	0.65	0.11	0.06	0.06	0.60
HDBSCAN	0.32	0.09	0.27	0.27	0.46	0.64	0.07	0.04	0.04	0.66
DPC	0.33	0.02	0.15	0.13	0.33	0.53	0.00	0.00	0.00	0.53
GMM	0.57	0.45	0.55	0.55	0.59	0.52	0.00	0.00	0.00	0.52
FCM	0.45	0.23	0.34	0.34	0.43	0.64	**0.15**	**0.12**	**0.12**	0.59
DPCSA	0.53	0.57	0.35	0.35	0.51	0.64	0.03	0.01	0.03	0.70
SC	0.32	0.08	0.25	0.25	0.46	0.64	0.00	0.00	0.00	0.71
AP	0.25	0.20	0.51	0.50	0.34	0.06	0.01	0.08	0.06	0.14
LW-DPC	0.68	0.43	0.56	0.58	0.59	0.62	0.03	0.05	0.00	0.62
**Algorithms**	**Spambase**					**WPBC**				
	**ACC**	**ARI**	**NMI**	**AMI**	**FMI**	**ACC**	**ARI**	**NMI**	**AMI**	**FMI**
DPC-DG	**0.61**	0.00	0.01	0.01	**0.72**	**0.67**	0.02	0.02	**0.02**	**0.68**
K-means	0.60	0.00	0.01	0.01	0.72	0.60	**0.03**	0.03	0.02	0.58
HDBSCAN	0.51	0.05	0.14	0.13	0.58	0.55	0.01	0.00	0.01	0.57
DPC	0.56	0.08	0.07	0.07	0.68	0.65	0.02	0.00	0.00	0.65
GMM	0.57	0.01	0.00	0.00	0.55	0.58	0.02	0.03	0.02	0.57
FCM	0.57	0.02	0.01	0.01	0.52	0.51	0.00	0.00	0.00	0.56
DPCSA	0.60	0.00	0.01	0.01	0.65	0.63	−0.03	0.01	0.01	0.68
SC	0.52	0.02	0.03	0.04	0.54	0.66	0.01	0.00	0.00	0.68
AP	0.33	**0.27**	**0.56**	**0.54**	0.47	0.13	0.00	**0.06**	0.02	0.20
LW-DPC	0.54	0.05	0.52	0.47	0.69	0.62	0.00	0.01	0.02	0.58

As shown in Table 4, on the German dataset, DPC-DG outperforms the other algorithms in terms of ACC and FMI, while K-means performs well in terms of ARI. AP leads in terms of NMI, while FCM shows a higher AMI score. These results show that DPC-DG is able to effectively capture the true cluster structure, especially in terms of compactness and consistency, while K-means performs well when the clusters are well separated. On the Liver dataset, DPC-DG again performs well in terms of ACC and FMI, while DPC achieves higher scores in terms of ARI, NMI, and AMI. This highlights that DPC-DG is able to accurately assign data points to clusters based on local density patterns, while DPC relies more on manual parameter settings, which may not always be optimal. For the Landsat dataset, DPC-DG performs well in terms of ACC, ARI, NMI, and FMI, demonstrating its robustness in handling datasets with different cluster shapes and densities. In contrast, K-means performs better on AMI but is weaker in capturing the intrinsic structure of the data. On the Pima dataset, DPC-DG outperforms other algorithms on ACC and FMI, while FCM performs better on ARI, NMI, and AMI. This further highlights the advantage of DPC-DG over the average-based FCM method in providing more accurate clustering solutions based on density peaks. For the Spambase dataset, DPC-DG achieves the best results on ACC and FMI, while AP performs well on ARI, NMI, and AMI, indicating that DPC-DG is particularly suitable for datasets with complex clustering structures. Finally, on the WPBC dataset, DPC-DG performs well on ACC, AMI, and FMI, while K-means achieves a higher ARI score. AP performs well on NMI, highlighting the different advantages of these algorithms on different evaluation metrics.

Despite its impressive performance on real-world datasets, DPC-DG still faces challenges in high-dimensional spaces, primarily due to the curse of dimensionality. As the number of dimensions increases, the adjacency relations constructed using Delaunay triangulation may become less precise, leading to misclassifications, especially when the data points are sparse or the dataset has high variance. In these cases, the domino effect may cause neighboring data points to be incorrectly assigned to different clusters. While DPC-DG remains competitive in identifying clusters in high-dimensional spaces, further optimizations are needed to address these challenges, such as improving the robustness of Delaunay triangulation in high-dimensional spaces.

## 5. Conclusion

In this paper, we proposed DPC-DG, a novel density peak clustering method based on Delaunay triangulation, designed to address the limitations of traditional DPC. Experimental results demonstrate that DPC-DG can effectively identify clusters with arbitrary shapes, outperforming other clustering algorithms in most scenarios. The method’s ability to automatically determine the number of clusters and avoid manual parameter tuning makes it highly adaptable to various datasets. Future Work and Applications: Looking ahead, DPC-DG shows promising potential for real-world applications in areas such as image segmentation, biomedical data analysis, anomaly detection, and social network analysis, where identifying complex, non-convex clusters is crucial. Its ability to handle datasets with varying densities and complex structures positions it well for these tasks. However, some challenges remain, particularly in high-dimensional spaces and large-scale datasets. The curse of dimensionality could affect the accuracy of Delaunay triangulation, and high computational cost could be a concern for massive datasets. Future work will focus on optimizing the algorithm to address these issues, including developing more efficient techniques for constructing the Delaunay graph and improving its performance in high-dimensional and large-scale settings. Additionally, integrating deep learning techniques to enhance DPC-DG’s scalability and robustness could further broaden its applicability. By addressing these challenges, we believe that DPC-DG can become a powerful tool for clustering complex and high-dimensional data in various domains, paving the way for more accurate and efficient data analysis methods.

## Supporting information

S1 File The supplementary datasets and additional materials referenced in this study are publicly available through the GitHub repository hosted at: https://github.com/7ucK1n9/S1-Data.git
(ZIP)
